# Population Epigenetics: The Extent of DNA Methylation Variation in Wild Animal Populations

**DOI:** 10.3390/epigenomes6040031

**Published:** 2022-09-28

**Authors:** Valentine Chapelle, Frédéric Silvestre

**Affiliations:** Laboratory of Evolutionary and Adaptive Physiology, Institute of Life, Earth, and Environment, University of Namur, 61 Rue de Bruxelles, 5000 Namur, Belgium

**Keywords:** population epigenetics, DNA methylation variation, epimutation, natural animal populations, evolution

## Abstract

Population epigenetics explores the extent of epigenetic variation and its dynamics in natural populations encountering changing environmental conditions. In contrast to population genetics, the basic concepts of this field are still in their early stages, especially in animal populations. Epigenetic variation may play a crucial role in phenotypic plasticity and local adaptation as it can be affected by the environment, it is likely to have higher spontaneous mutation rate than nucleotide sequences do, and it may be inherited via non-mendelian processes. In this review, we aim to bring together natural animal population epigenetic studies to generate new insights into ecological epigenetics and its evolutionary implications. We first provide an overview of the extent of DNA methylation variation and its autonomy from genetic variation in wild animal population. Second, we discuss DNA methylation dynamics which create observed epigenetic population structures by including basic population genetics processes. Then, we highlight the relevance of DNA methylation variation as an evolutionary mechanism in the extended evolutionary synthesis. Finally, we suggest new research directions by highlighting gaps in the knowledge of the population epigenetics field. As for our results, DNA methylation diversity was found to reveal parameters that can be used to characterize natural animal populations. Some concepts of population genetics dynamics can be applied to explain the observed epigenetic structure in natural animal populations. The set of recent advancements in ecological epigenetics, especially in transgenerational epigenetic inheritance in wild animal population, might reshape the way ecologists generate predictive models of the capacity of organisms to adapt to changing environments.

## 1. Introduction

Our understanding of an organism’s capacity to respond to environmental changes has advanced, in a large way, through studies focusing on genetic variation and the manipulation of environmental conditions. These studies confirm that genotype, environment, and their interaction contribute to phenotypic variability, a fundamental prerequisite for evolution by natural selection. The tremendous development of genetic knowledge during the 20th century has led to the merge of Darwinism and the field of genetics into a modern synthesis. However, we now admit that genetic variation is not the only source of phenotypic variation that can be inherited across generations because only a small proportion of variance in complex traits can actually be explained by genetic variance [1]. The concept of inclusive heritability has been proposed to unify genetic and non-genetic mechanisms of heritability, which encompasses all dimensions of inheritance such as the transmitted parental effect, ecological variation, social variation, and transgenerational epigenetic inheritance (TEI) [2]. There is a growing consensus that epigenetics and, in particular, TEI could be one of the missing factors for understanding phenomena that cannot be explained by the DNA sequence alone, such as incomplete penetrance (i.e., individuals of a given genotype expressing different phenotypes) and the variance in expressivity (i.e., the degree/intensity to which complex trait expression differs among individuals) [3,4]. These two phenomena result in an incomplete correlation between genotype and phenotype, and these may be partly explained by epigenetic mechanisms.

Epigenetics has been narrowly defined as mitotically and/or meiotically heritable changes in gene expression that cannot be explained by changes in the gene sequence [5] (see Table A1 for glossary). These changes include histone modification, DNA methylation, and small RNA regulation, and these are involved in processes such as cellular differentiation, development, diseases, behaviors, and metabolism [6]. Studies exploring phenotypic plasticity have showed that epigenetic variation can play a significant role in the response that an organism has to environmental variation, as epigenetic marks can be directly affected by the environment. In other words, environmentally induced epigenetic variations have been proposed to mediate phenotypic plasticity as they allow the organisms to adapt to the environmental conditions by increasing the phenotypic options of a genotype with no genetic sequence modification [7,8,9]. Moreover, fitness-related phenotypes that are initially environmentally induced can be selected to become genetically determined, and hence, heritable, a process that is named genetic accommodation [10,11]. Genetic assimilation (i.e., the loss of or decreased plasticity [12]) and genetic compensation (i.e., the selection for similar phenotypes in different environments [13]) are different types of genetic accommodation. In other words, in addition to being another source of phenotypic variation, epigenetic variation can precede genetic adaptation through genetic accommodation, thus reversing the standard model of evolution from a genotype-to-phenotype to a phenotype-to-genotype information flow [11,14,15].

Understanding the evolutionary implications of epigenetics and how epigenetic mechanisms contribute to phenotypic variability is one of the current greatest challenges in evolutionary biology. The importance of epigenetic variation in environmental adaptation and evolution has been investigated much more in plants than it has been in animals [16,17]. Moreover, most epigenetic data that are available on animals have been collected under laboratory conditions in model organisms such as mice and insects and these studies have mainly focused on epigenetic mechanisms and their responses to environmental stressors [17,18,19,20]. Laboratory studies on plants and animals have shed light on some of the general features of epigenetics, with important evolutionary implications. First, epimutations were assessed to be up to five orders of magnitude more frequent than genetic mutations were (10^−4^ versus 10^−9^ per base pair and generation) in the model plant *Arabidopsis thaliana* [21,22]. Second, some epigenetic marks may be stably inherited across generations through transgenerational epigenetic inheritance, as reported in many plant and animal taxa such as mammals [23], birds [24], fish [25], and invertebrates [26,27]. Third, epigenetic variation that is associated with changes in gene expression can be environmentally induced in plants and animals [28,29].

An important step is now to examine the extent of epigenetic variation and the way that this variation changes over time in wild populations that encounter natural levels of environmental complexity, genetic structure and dynamics, and natural ecological processes. This endeavor represents part of the field of population epigenetics. Though the basic concepts of population genetics from the 1930s are well described, these have been extended with the introduction of the modern synthesis (MS), however, the body of knowledge concerning population epigenetics remains largely scarce as it is a new research interest. As for laboratory studies, most of the natural population, epigenetic research projects have been carried out in plant populations, which have been reviewed elsewhere [16,30,31]. The first experimental work investigating epigenetics in natural animal populations was published in 2010 on DNA methylation in rainbow trout *Oncorhynchus mykiss* [32]. Since this study, there has been a growing number of studies focusing on natural population epigenetic variation, especially DNA methylation variation in animals in terms of their phenotypic diversity generation and local adaptation. 

As is the case for most of these articles, the present review focuses on DNA methylation, which is the most extensively characterized epigenetic mechanism in both plants and animals [33]. DNA methylation is found across all taxa of life, and primarily occurs at the 5-methylcytosine bases in eukaryotes and prokaryotes [34]. It involves the addition of a methyl group to cytosine within the CpG dinucleotides in animals. The DNA methylation of regulatory regions is generally associated with gene down-regulation or silencing, but that is not always the case [35,36]. Recent studies have showed that gene body methylation is positively correlated with transcriptional activity in most animal species [33]. The genomic distribution of DNA methylation has been described in many clades of animals, but there are some differences in how and where it occurs. In vertebrates, the pattern and extent of DNA methylation is well conserved across species; DNA methylation occurs nearly throughout the entire genome, with 70–80% of cytosines in the CpG dinucleotides being methylated [37]. Gene bodies, including exons and introns, are typically methylated, while CpGs in the gene promoter regions are often lowly methylated [38,39]. The idea that only vertebrates have a highly methylated genome has recently been challenged as this phenomena has also been found in the sponge *Amphimedon queenslandica* and a unicellular green algae from the genus *Chlorella* [34,40]. Despite this rather consistent DNA methylation pattern across vertebrate species, differences occur in terms of the pattern establishment during early embryogenesis. Taking DNA methylation reprogramming as an example, the demethylation of both parental genomes occurs in the mouse embryo, whereas the paternal pattern of methylation is maintained in zebrafish, with a reprogramming of the maternal DNA to correspond to the paternal template [41]. Regarding invertebrates, DNA methylation patterns are extremely variable across taxa. Some invertebrate genomes lack cytosine methylation such as the nematode *Caenorhabditis elegans*, the platyhelminth Schmidtea mediterranea, the fruit fly *Drosophila melanogaster*, and the rotifer *Adineta vaga* [42,43], while others are similar to plants as they have a mosaic of heavily methylated DNA domains (predominantly in exons) that are interspersed with domains that are methylation-free, such as the sea anemone, honey bee, and sea squirt [34,44]. Although the DNA methylation pattern and its genomic distribution vary widely across animal taxa, it is possible to draw general lines on its diversity and its responsiveness when it is facing natural environmental conditions. 

Our discussion starts with a presentation of existing literature on DNA methylation variation and genetic variation within and among natural animal populations. It then focuses on the relationship between epigenetic and genetic variation, which illustrates a degree of autonomy of DNA methylation variation from genetics, and ultimately, its additional value in evolutionary mechanisms. The following section describes the epigenetic dynamics in natural animal populations. Some ecological processes act on epigenetic variation and patterns, and others act on both epigenetic and genetic structures. It is crucial to consider these processes to understand the current patterns of genetic and epigenetic variation, but also the past and the future populations’ epigenetic dynamics. Then, we discuss the extended theory of evolution, including epigenetic variation as an evolutionary mechanism in natural populations. Epigenetic variation may be involved in population microevolution (rapid evolutionary events that are adaptations to a new environment during introduction and invasive events in fast-changing habitats and when stressors are occurring), but also in population macroevolution, including radiation and speciation. The review closes with a discussion on the directions of future studies on the epigenetics of wild animal populations. Addressing these topics is essential to achieve a more comprehensive understanding of the relevance and the roles of epigenetic mechanisms, especially DNA methylation, in regulating phenotypic plasticity and facilitating evolution in wild animal populations.

## 2. Epigenetic Diversity in Natural Animal Populations

DNA sequences are a succession of four different bases (A, C, T, and G), and each mutation switches a base for another one. An allele is a variant of the same gene that is located at the same genetic locus and is characterized by a specific sequence. Each diploid organism owns two alleles at each locus, and it is qualified as heterozygous if the alleles are different, or homozygous if they are the same. The situation is quite different for DNA methylation marks, since a cytosine can only be methylated (M) or unmethylated (U), thus restricting to two the number of possibilities that there can be at each cytosine. For the same allele, each CpG (and at a lower level, each CHG and CHH-H for any base, except for G) can either be M or U, thus producing a succession of single methylation polymorphisms (SMPs). SMPs can accumulate in the DNA sequence, and they produce a specific methylation pattern, or epiallele. While genetic variation refers to the different allele frequencies that there are among individuals or populations, epigenetic variation corresponds to the presence or absence of epigenetic markers at specific loci that are studied among and/or within populations [45]. The amount of epigenetic variation within a population is called epigenetic diversity, and it refers to SMP diversity [46]. SMP diversity is generated by epimutations, i.e., epigenetic modifications at a given position or region, and its origins can be genetic, environmental, or stochastic [45,47]. Focusing on cytosine methylation (5mC), epimutations are heritable changes in the methylation status of a single cytosine or of a region or cluster of cytosines [48]. To determine the DNA methylation diversity in wild animal populations, most field studies have used methylation-sensitive amplified polymorphism (MSAP or MS-AFLP) [49,50,51]. Next-generation sequencing is, more rarely and most recently, the method that has been used among other such as reduced representation bisulfite sequencing (RRBS) [52,53,54,55], MeDIP-Seq analysis [56,57], and whole-genome bisulfite sequencing (WGBS) [58] (Table A2). 

By comparing the epigenetic and genetic diversity in wild animal populations, it is possible to estimate the relative importance of genetic and epigenetic variation in populations phenotypic diversity, and to test the hypothesis that epigenetic divergence acts as the first step in speciation, allowing for the expression of alternative phenotypes in response to environmental changes, which are ultimately fixed by genetic accommodation or assimilation [11,15,59,60]. Many studies have identified extensive epigenetic diversity that exceeds the genetic diversity between natural populations of plants [61,62]. It was suggested that the epigenetic variation in natural plant populations plays a major role for their transient and/or heritable adjustment to the changing environments, as it may be stable and related to environmental variation [63]. This implies that these environmentally induced epimutations may lead to the convergence of individuals that are living in similar habitat conditions; this is a situation that may be exacerbated by TEI. Despite the fact that studies on epigenetic variation in natural animal populations are scarce when they are compared to plant studies, some discernible patterns have emerged after we have reviewed them. As result of our review, regardless of whether we focused on crustaceans, mollusks, fish, reptiles, birds, or mammals, the DNA methylation variation was larger than the genetic variation was among and/or within wild animal populations [53,56,64,65,66,67,68]. For example, Smith et al. studied the DNA methylation variation in fish (*Etheostoma olmstedi*) using the MSAP technique. They investigated two North American river drainages, wherein, each of them include several closely related populations, to characterize the epigenetic variation within and among the populations. They obtained results that demonstrated that there is a significantly greater epigenetic diversity than there is genetic diversity within all of the populations in both the Patuxent and Potomac rivers. Regarding the diversity among the populations, their analysis demonstrated that there is a substantial epigenetic structure, but no genetic structure, meaning that *E. olmstedi* populations are significantly different from each other in terms of their DNA methylation patterns, but not in terms of their genomes [60]. They assumed that the methylome is changing faster than the genome is in this species, which is in accordance with the general hypothesis that epigenetic divergence can precede genetic divergence in evolution due to its dynamics.

A larger amount of epigenetic diversity in comparison to the amount of genetic diversity can also arise between populations of sister species. Skinner et al. measured the genetic mutations (via copy number variation—CNV) and epimutations (via differential DNA methylation regions—DMRs) across five species of Darwin’s finches (*Geospiza fuliginosa*, *G. fortis*, *G. scandens*, *Camarhynchus parvulus*, and *Platyspiza crassirostris*). As a result of these measurements, they found that there were fewer genetic mutations than there were epimutations among the five species, showing that the differences in the methylome are more related to evolutionary relationships than they are differences in the genome. Moreover, they reported that the differentially methylated genes were related to evolutionarily important pathways in birds [65]. Vernaz et al. found a substantial methylome divergence between six Lake Malawi cichlid species that show extensive phenotypic diversity despite having them extremely low DNA sequence divergence. These DMRs were enriched in transposons and were associated with the transcription changes of ecologically relevant genes that are related to energy expenditure and lipid metabolism in the cichlid’s liver [68]. 

An extreme situation can be observed in asexual species exhibiting a lack of genetic variation due to their reproductive system. A study on epigenetic polymorphism in the clonal fish *Chrosomus eos-neogaeus* from seven geographically distant lakes showed that they have an interindividual DNA methylation variability. Moreover, individuals could be regrouped according to their lake of origin on the basis of their unique methylation profile, as individuals of a given lake are epigenetically similar [69,70]. Thorson et al. measured the genome-wide DNA methylation variation of asexual New Zealand freshwater snail *Potamopyrgus antipodarum* from distinct habitats (two lakes versus two rivers). Those snails have significant methylation signatures when one is comparing those of the lake versus those of the river populations [71]. Later, they examined the methylation variation among those in the lakes that differ in their environmental disturbance and pollution histories. Using an MeDIP-Seq analysis, they showed the presence of site-specific differences in the DNA methylation between each of those lake populations [57]. These studies raise the question of the environmental implications in epigenetic variability, which is discussed later.

In most of the wild animal populations that have been examined to date, independently of the studied organisms or the molecular analysis that is being used, the DNA methylation variation is larger than the variation in allele frequencies within and among natural animal populations. This should not be surprising as epimutations can happen randomly, such as mutations, but they can also be triggered by environmental conditions and by the genotype itself. The epigenetic diversity that is found in a population is therefore the result of the combination of these three distinct sources. To determine the implications of the epigenetic processes in evolution, a major concern is to characterize the degree of autonomy between epigenetic and genetic variation and ultimately, the degree of phenotypic variation that can be explained only by the environmental or stochastic epimutations [72].

## 3. Correlation between Epigenetic and Genetic Variation in Natural Animal Populations

Based on their degree of autonomy from the underlying genotype, epialleles are categorized into three types: obligatory, which is completely dependent on the genetic variation; facilitated, which is directed or loosely potentiated by the genotype; pure, which is independent of the genetic variation and is generated by stochastic events or environmental changes [73]. To identify which epialleles categories are encountered in natural populations, the correlation between the genetic and epigenetic profiles can be estimated, mostly by using a Mantel test [50,63,74,75]. A significant (positive or negative) correlation suggests that the epigenetic and genetic variations are interdependent, which corresponds to the presence of obligatory epialleles. In contrast, the absence of a significant correlation suggests that the epigenetic variation can autonomously impact the phenotypic variation, by being totally or partly independent from genetic control.

Under laboratory conditions, epimutations are expected to be mostly obligatory. The lack of environmental fluctuations in the laboratory housing conditions does not promote environmentally induced epimutations and the selection of epimutation-sensitive alleles that are responsible for alternative phenotypes that occur while experiencing environmental changes in natural conditions (see the Baldwin effect in “Section 5.2 Epigenetics and macroevolution of natural animal population”). In this case, epigenetic variation can be viewed as a phenotypic read-out downstream of the genotype, with a low environmental contribution. For example, Hu and Barrett reviewed the epigenetically encoded thermal plasticity in animals. Of the 14 studies, 13 included a putatively obligatory epigenetic variation that was underlying phenotypic plasticity, and only one was categorized as “unknown” [45]. In mice and humans, some studies have evaluated the association between epigenetic and genetic variation with a narrow-sense heritability, i.e., the ratio of additive genetic variance to the total phenotypic variance. It appears that genetic variation can explain an average of 7-34 % of all methylation variation [76,77,78]. In natural animal populations, although several studies have measured epigenetic and genetic variation, only a few of them have estimated the relationship between those variations. Of the 26 reviewed studies, 12 did not calculate a correlation coefficient between the genetic and epigenetic variation, eight studies found a non-significant correlation, and six studies obtained a significant correlation (Table 1). Moreover, few authors have linked a calculated correlation coefficient to Richards’ epiallele categories. For instance, Liebl et al. obtained a significant negative correlation between the genetic and DNA methylation variation within seven populations of house sparrows (*Passer domesticus*) [79]. However, they predicted that all three kinds of epialleles could play a role in those populations as their design could not discriminate between the three categories. Despite the lack of direct connection between these categories and the genetic vs. epigenetic variation–correlation coefficient, calculating this coefficient can still help to estimate the relative importance of the genetic and epigenetic variation in the mechanisms that are facilitating population divergence, and to highlight the extent to which epigenetic variation is under genetic control [67,73]. 

Despite the fact that the obligatory epialleles’ relevance is questioned regarding its evolutionary potential [69], an interesting new insight is that the epigenome can also influence the genome, thereby creating a significant relationship between their variation. Firstly, the epigenetic variation can regulate the active status of transposable elements (TEs). TEs are DNA sequences that have the ability to change their position within a genome. The epigenetic control of gene expression mostly originates from the regulation of TEs that are inserted near genes [83]. In fact, TEs are the major carriers of epigenetic marks and are subject to almost all epigenetic regulatory mechanisms in plants [84]. Interestingly, there is a great variability in the locations of the TEs, not only between different species, but also within populations. More than 90% of the TEs that are inserted at a specific genomic position are not present in all individuals within both animal [85] and plant [84] populations. Transposon insertion polymorphism between individuals may come from epigenetic variation and it results in genomic sequence variation. Secondly, DNA methylation variation can influence the mutation rates in repetitive elements which are known to regulate genome 3D folding and the establishment of heterochromatin, among other regulation mechanisms [86]. These repetitive elements are patterns of nucleic acids that occur as multiple copies in the DNA sequence, comprising TEs, simple sequence repeats (SSRs), and microsatellites, and these represent a major fraction of vertebrate genomes [87]. Some studies have highlighted the correlation between a decrease in the DNA methylation of specific repetitive elements and an increase in the copy number variations (CNVs), thus showing another possible epigenetic-to-genetic flow [88,89]. Finally, besides these specific genome components, epimutations may alter the global genome stability and modify the mutation rate of the DNA sequences throughout the genome. Methylated cytosines (mCs) in the CpG context seem to have a higher mutation rate than non-methylated ones [90], and this is in part because mCs are subject to spontaneous deamination [91]. This process, where an mC turns into a T, occurs at a rate that is 10- to 50-fold higher than any other mutation is in humans [92]. The result of this hypermutability is a CpG depletion in the consistently methylated genomic regions. Yet, the amount of CpGs in a genome partly determines its epigenetic potential, which is defined as “the capacity for environmentally induced phenotypic change (i.e., plasticity) via epigenetic modifications to relevant genomic elements” [93]. CpGs are considered as the capacitors of phenotypic plasticity; the more CpGs an organism has, then the more facilitated their acclimation is via DNA methylation and gene expression regulation.

**Figure 1:**
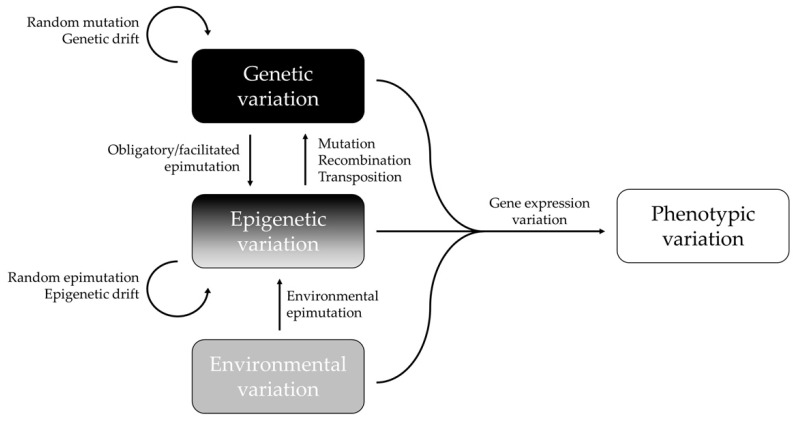
Interactions between epigenetic, genetic, environmental, and phenotypic variations. Epigenetic variation can depend upon the genotype (obligatory and facilitated epimutations), or it can be independent of the genotype (pure epimutations) and be generated by environmental changes or stochastic events (random epimutation/epigenetic drift). Adapted with permission from [47], 2022, Frédéric Silvestre.

In summary, epigenetic and genetic changes most likely work in concert to regulate the gene expression and phenotypic variation of complex traits (Figure 1). The proportion of the genotype-independent and -dependent epigenetic variation reflects the underlying mechanisms of the natural animal population’s evolutionary pathways to promote phenotypic variation [65]. New insights that we would like to highlight is that even though there is a significant correlation between epigenetic and genetic variation, epigenetic variation is not necessarily dependent on genetic variation. As has already been explained, the epigenome can influence the genome in different ways. Moreover, a significant correlation does not imply that there is a causal relationship. Geographical and ecological processes may create parallel evolutions of genetic and epigenetic structures, and thus, similar patterns that are without any functional link with each other. To better understand these dynamics, it is important to consider the mechanisms that are influencing both genetic and epigenetic diversity, and the processes that only act on epigenetic markers in wild animal populations.

## 4. Epigenetic Dynamics in Natural Animal Populations

### 4.1. Geographical and Ecological Processes Acting on Both Epigenetic and Genetic Diversities

If the epigenetic marks are stably inherited, similar processes that contribute to generating patterns of the genetic structure in natural populations can also act on the epigenetic variation [54,57]. Gene flow is an important mechanism for transferring alleles between populations, thus resulting in increasing the homogeneity among populations, and in increasing the genetic diversity within a population. Some factors can decrease the gene flow, thus generating genetic isolation and in some case, speciation. The gene flow is reduced in the species with low dispersal or mobility, that are living in fragmented habitats, are made of small populations, and are separated by a long distance. This geographically limited dispersal creates genetic differentiation, which is also called isolation by distance (IBD) [94]. Herrera et al. proposed a similar approach to measure the epigenetic IBD among individuals or among populations, and to use the spatial structure of their genetic diversity as a null model to investigate the processes that are shaping epigenetic variation in natural populations [95]. The variable level of transgenerational epigenetic inheritance and the capacity of epigenetic marks to be modified in response to environmental variation are the two factors explaining the possible differences between genetic and epigenetic diversity. In plants, this approach generally shows that there is a greater level of epigenetic IBD than genetic IBD, and there is a higher epigenetic similarity when this is compared to the amount of genetic similarity at the shortest distance, suggesting that both a significant TEI and a high responsiveness to the environmental local conditions are the major drivers of epigenetic spatial structure [95]. 

Besides the geographical distance, the ecological conditions are other landscape elements that can influence gene flow. Temperature, precipitation, humidity, elevation, substrate type, and vegetation density are all examples of the environmental factors that can also play a role in evolutionary processes like isolation by environment (IBE). IBE is defined as a pattern in which the degree of genetic differentiation increases with the environmental differences, independent of the geographic distance [96]. A variety of processes can generate genetic IBE, including natural and sexual selection against migrants from divergent environments and biased dispersal. IBD and IBE, besides acting on genetic structure, can also act on epigenetic diversity as epialleles should be transferred between populations with gene flow [95]. DNA methylation divergence, genetic divergence, and reproductive isolation were investigated in eight pairs of geographically isolated species *Etheostoma* (‘darters’), a diverse genus of North American freshwater fish [60]. The strongest reproductive barrier among darter species seems to be the behavioral reproductive isolation, i.e., a reduction in gene flow due to differences in mating behavior [97]. They found a significant relationship between behavioral isolation and interspecific epigenetic divergence, but not with genetic divergence. These results suggest that the strength of the behavioral isolation among the eight allopatric, phylogenetically independent species is predicted by epigenetic divergence [60]. Another study reported significant DNA methylation differentiation that is consistent with short-distance dispersal among great roundleaf bat populations in China [98]. These studies illustrate the strong relationship that may exist between epigenetic and isolation mechanisms as gene flow reduction occurs due to sexual selection or dispersal capacity, thus creating a higher epigenetic population structure than that of the genetic structure. A recent study compared spatial genetic and epigenetic variation based on single nucleotide polymorphisms (SNPs) and single methylation variants (SMVs) from eight populations of the Puerto Rican crested anole *Anolis cristatellus* that occupies a diverse range of habitats [54]. They found that the plots of the genetic and epigenetic IBD and IBE indicate that they have similar slopes, suggesting that the genetic and epigenetic variation may have shared responses to geographical and environmental factors. Interestingly, after controlling for the effects of the underlying genetic structure, there is still a relationship between the epigenetic and genetic structure, but they did find evidence for a strong pattern of genome-wide epigenetic IBE. This significant epigenetic IBE suggests that the epigenetic variation in *A. cristatellus* is not only attributable to the pattern of genetic variation, but that epigenetic differentiation is strongly correlated with environmental divergence. This is the first study of its kind, as the empirical demonstration of epigenetic IBE has been limited to only a few plant systems [63,95]. This difference between genetic and epigenetic IBE probably arises from the ecological processes that influence epigenetic but not genetic diversity.

### 4.2. Ecological Processes Increasing Epigenetic Diversity

Some processes can act on epigenetic but not genetic variation, thereby contributing to the extensive epigenetic diversity that exceeds that of the genetic variance, as described above. These pure methylation variations may be created by stochastic events (like random epimutations or epigenetic drift) and are also induced by environmental variation. 

Random epimutations can arise at any time in the lifespan of the organism, and they are not induced by environmental factors. Accurately setting, erasing, and reproducing methylation patterns are complex processes involving a series of interconnected factors (reviewed in [47]). A first possible mechanism sustaining random epimutation refers to the imperfect fidelity of methylation replication. In eukaryotes, DNA methylation replication is catalyzed by the enzymes of the DNMT (DNA methyltransferase) family. DNMT1, also named as “the maintenance DNA methyltransferase”, has an accuracy rate of ~95%, despite its regulatory mechanisms such as autoinhibition [47]. This defect of 5% inaccuracy can generate new DNA methylation patterns, especially since replication is required over the entire genome. A second mechanism underlying the random epimutations is *de novo* methylation during early-life stages. Epigenetic marks are placed at very specific times during the organism’s development, namely gametogenesis and early embryogenesis. The patterns are set by other members of the methyltransferase family, namely DNMT3A and DNMT3B, which are also known as *de novo* methyltransferases. Due to mechanisms such as the imprinting of primordial germ cells and DNA methylation reprogramming, gametogenesis and early embryogenesis offer another window of susceptibility for random epigenetic alterations. Studies focusing on quantifying the epimutation rates have mainly focused on the model plant *Arabidopsis thaliana* [21,22,99]. The forward and backward CpG epimutation rates (i.e., methylation is gained or lost, respectively) were estimated to be 2.56 × 10^−4^ and 6.30 × 10^−4^ per generation per haploid methylome, respectively [22]. These estimates are similar to the rate that has been provided previously (4.46 × 10^−4^ by [21]), but they illustrate that methylation loss at the CpG is globally three times as likely as the methylation gain is. They also detailed the extent to which CpG epimutation rates depend on the genomic context, with the highest rates being found in gene bodies (forward: 3.48 × 10^−4^ and backward: 1.47 × 10^−3^), and the lowest rates being found in transposable elements (forward: 3.24 × 10^−4^ and backward: 1.20 × 10^−5^). Interestingly, a spontaneous error rate in methylation maintenance at the promoter CpG islands (both gains and losses) was measured to be 10^−4^ to 10^−5^ in vitro [100], which means that even the genome regions that are essential in gene expression regulation can be modified by random epimutations. This set of results contrasts with the spontaneous genetic mutation rate of 7 × 10^−9^ base substitutions per site per generation in *A. thaliana*, 2.3 × 10^−10^ in *C. elegans*, 3.4 × 10^−10^ in drosophila, and 5.0 × 10^−11^ in humans [101,102]. In other words, random epimutations can emerge at any time in the lifespan of the organism, with rates that are expected to be higher than the genetic mutation rates. Some events have a high susceptibility for random epigenetic alterations including cell division, gametogenesis, and embryogenesis.

Epigenetic drift corresponds to the gradual changes in epigenetic patterns, and it is due to random epimutations. This neutral process is not directional as it creates both hyper- and hypomethylation. Moreover, drift is not uniform across the genome, and is variable between individuals of the same age. A meaningful drift example is age-related epigenetic drift. This uncoordinated accumulation of methylation variation creates a global DNA hypomethylation and degrades the transcriptional networks during aging [103]. This process is variable across the genome, may not occur homogeneously in all cells, and is variable between individuals of the same age. Thus, epigenetic drift leads to increased discordance between individual epigenomes across the lifespan of the organism. Conversely, some programmed changes in the methylation of specific CpG sites are consistently related to age between individuals of the same species. This programmed aging-associated epigenetic modification refers to the epigenetic clock [104]. The prevailing tendencies of these specific changes are the hypermethylation of the promoter sequences that are associated with CpG islands and the hypomethylation of CpG-poor genes. There is a strong correlation between the age and methylation levels of multiple CpG sites in individuals of the same species [103], whereby, their methylation status could be used as an epigenetic signature to estimate their biological age. Until recently, this “epigenetic clock” had only been developed in mammals, including humans, mice, whales, dogs, and wolves. A large international consortium recently compared thousands of methylation marks among 59 tissues and constructed highly accurate universal epigenetic clocks for 128 mammalian species [105]. Although very little is known about non-mammalian vertebrates, recent studies have also relied on DNA methylation repatterning during aging to develop such epigenetic clocks for a few fish species, including zebrafish, Japanese medaka, European seabass, Australian lungfish, Murray cod, and Mary River cod [106,107]. Thus, both epigenetic drift and the epigenetic clock contribute to time-related changes in DNA methylation, but in fundamentally different ways. In both cases, gene expression regulation by epigenetic mechanisms becomes gradually deregulated therefore leading to a diminished responsiveness to environmental stimuli. Ultimately, epigenetic drift could lead to a loss of cellular phenotypic plasticity [108]. Studies focusing on whether and how drift influences epigenetic marks in wild animal populations are very scarce. Recently, Venney et al. provided new evidence that drift could act on DNA methylation by highlighting the correlation between microsatellites (considered as neutral genetic markers of genetic drift) and the differences in methylation among eight populations of Chinook salmon (*Oncorhynchus tshawytscha*) [82]. Despite there being a lack of studies focusing on epigenetic drift and random epimutations in wild animal populations, several field studies have explained the presence of large amounts of epigenetic diversity in contrast with the presence of smaller amounts of genetic diversity with the occurrence of mechanisms including stochastic epigenetic drift and epimutations in a wide range of animals [54,57,60,65,66,67,71,81]. 

Beside these processes, which are similar to those of genetics (genetic/epigenetic drift and random mutations/epimutations), another major mechanism can act specifically on epigenetics markers: environmentally induced epimutations. Unlike genetic variation, DNA methylation can be rapidly influenced by environmental variation, particularly when the organism is in the early developmental stages [109]. Some studies have investigated the influence of the environment in shaping the epigenome under different laboratory settings (e.g., temperature [110], diet [111], behavior [112], and chemicals [113]. However, they may not reflect the epigenetic processes that occur under field conditions with natural levels of environmental heterogeneity and complexity. Field studies on plant populations have shown that there are strong environmental effects on DNA methylation [30,63,95,114]. Similar results have been obtained for animals as population epigenetic studies have provided evidence of habitat-specific DNA methylation patterns in a wide range of wild animal species (e.g., [50,53,54]), especially between ecotypes, such as freshwater vs. marine three-spined sticklebacks *Gasterosteus aculeatus* [115], coastal vs. offshore common bottlenose dolphins *Tursiops truncates* [116], and lake vs. stream ecotypes of clonal fish *Chrosomus eos-neogaeus* [80]. These observations of environmentally induced epimutations are even more likely in habitats that are disturbed by urbanization and/or pollution, wherein DNA methylation variation could be driven by a variation in food availability and pollutant levels [55]. Guillette et al. have focused on the potential alterations in the epigenome of the American Alligator *Alligator mississippiensis* that live in contaminated (Lake Apopka—AP and Merritt Island—MI) and non-contaminated (Lake Woodruff—WO) lakes in Florida. They identified 85 differential DNA methylation regions between WO and AP, and 75 between WO and MI, showing that there are more epigenetic alterations in the species in the contaminated lakes compared to those in the species in the non-contaminated lake [109]. Similar results have been observed between asexual snails *Potamopyrgus antipodarum* living in rural lakes vs. urban lakes [57], between hatchery and natural-origin steelheads *Oncorhynchus mykiss* [52], between baboons *Papio cynocephalus* that forage naturally in a savanna environment vs. baboons that have access to spatially concentrated human food scraps [117], and also between two closely related species of Darwin’s finch living in rural vs. urban populations [56]. Regarding this last study, more interestingly, few of the DMRs between the rural and urban populations were found in the same DNA sequence regions in *G. fortis* and *G. fuliginosa*. This suggests that these species are responding to environmental changes in different ways, which correspond to a species-specific sensitivity to environmental variations, even when they were comparing the closely related species [56]. These studies show habitat-, population-, or species-specific DNA methylation patterns in a wide range of wild animal populations, indicating that local environmental factors may influence DNA methylation patterns among populations. Thus, environmentally induced epimutations could ultimately contribute to the extensive epigenetic diversity that is observed in the animal populations studies that are reported in this review. Moreover, the environmentally induced epigenetic variation between natural populations could be even greater in the case of isolation, especially isolation by environment, as described above. Ultimately, environmentally induced epimutations could lead to local adaptations if these marks are inherited across generations. 

To date, we have found very few studies that evaluated the transgenerational inheritance of DNA methylation marks in natural animal populations encountering different environmental conditions. Wang et al. investigated the environmentally induced phenotypic variation, DNA methylation, as well as heritable epigenetic variations between intertidal and subtidal Pacific oysters (*Crassostrea gigas*) using WGBS. Their offspring F1 were produced and subjected to a common environment. There was a clear DNA methylation differentiation between the intertidal and subtidal oysters, as they identified 3012 differentially methylated genes (DMGs) in F0, and 3090 DMGs in F1. Moreover, the 1238 DMGs that were found in the F1 oysters were shared with those in the F0 generation, meaning that about 41% of the DMGs between the intertidal and subtidal oysters could be transmitted to the next generation. They also investigated the variation tendency in the 1238 inherited genes. Nearly 70% of the heritable DMGs had a consistent variation trend in response to the environments in the two generations. Finally, these DMGs were annotated, and they appeared to be involved in phosphorus, lipid, and protein metabolism, and in the regulation of GTPase activity, autophagosomes, and apoptosis [58]. This study highlighted the inherited environmentally induced methylation variation that may underlie the phenotypic divergence that is related to the heat stress between intertidal and subtidal oysters across generations. A second study that was carried out by Hu et al. showed similar results by comparing the DNA methylation variation between marine and freshwater ecotypes of threespine sticklebacks (*Gasterosteus aculeatus*) using an RRBS technique. F0 fish were collected in marine and freshwater locations and maintained in a common garden. F1 and F2 subjects were generated by crossing the marine and freshwater wild-caught parents to explore their stable epigenetic variation and its underlying genetic basis across two generations of the marine-freshwater hybrid lines. Firstly, they identified 891 differentially methylated cytosines (DMCs) between the parental fish that were sampled from the marine versus the freshwater habitats. Then, they investigated the levels of intergenerationally stable methylation. They found that 94.8% (845 out of 891) of the DMCs between the ecotypes were identified as stable across generations, suggesting that this methylation divergence could play a role in facilitating their adaptation to different habitats. They also found a narrow-sense heritability of these stable DMCs, ranging from 24% to 35%, meaning that some of them are obligatory epimutations (under genetic control), while other are pure epimutations. Finally, their functional analysis identified several DMC-associated genes that are related to environmental variations such as salinity, osmosis, parasites, and diet [118]. Those two recent studies bring new insights into the extent to which variation in environmentally induced DNA methylation is stably transmitted across generations in wild animal populations, and they provide promising evidence for the adaptive mechanisms through which these transmitted epimutations occur.

**Figure 2:**
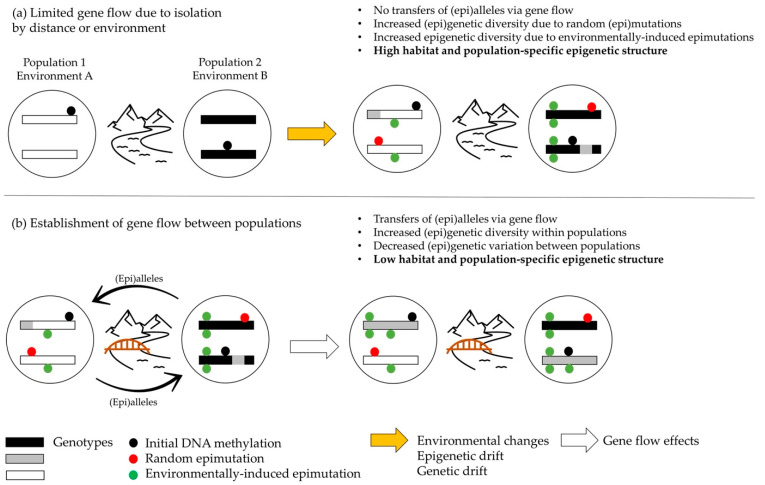
Epigenetic dynamics: ecological and geographical processes and their consequences on (epi)genetic diversity within populations and on (epi)genetic variation between populations. (**a**) Isolation by distance or by environment limits (epi)alleles transfer by gene flow. Stochastic events such as drift and (epi)mutations increase (epi)genetic diversity within populations over time. Environmentally induced epimutations create habitat and/or population-specific DNA methylation patterns that may be transmitted to the next generation, resulting in strong epigenetic structures. (**b**) As consequences of gene flow, (epi)genetic diversity increases within populations and (epi)genetic variation between populations decreases, thereby resulting in lower epigenetic structure that distinguished populations.

To summarize, a population’s epigenetic and genetic structures might be the consequences of the combination of ecological mechanisms that are in common with or specific to genetic and epigenetic dynamics (Figure 2). Gene flow can transfer both alleles and epialleles between different populations, thereby dealing with barriers such as geographical, environmental, or reproductive barriers. Stochastic events such as drift and mutations/epimutations also act on both genetic and epigenetic divergence, with there being a possible greater impact on epigenetic markers as the epimutation rates are expected to be higher than the genetic mutation rates are. A specificity of epigenetic markers is their responsiveness to environmental variation. It can create habitat-, population-, or species-specific DNA methylation patterns that may be transmitted to the next generation, resulting in among-population but also within-population variations, as individuals are likely to display different sensitivities to environmental stimuli. Taking these processes into account, the observed greater amount of epigenetic variability that is seen when this is compared to the amount of genetic diversity might be caused by epigenetic drift and random or environmentally induced epimutations, whose effects are exacerbated in the situation of limited or insufficient gene flow to prevent divergence. This situation mainly occurs in species with a low dispersal or mobility, those that are living in fragmented habitats, are made of small populations, and are separated by a long distance, thus promoting genetic and epigenetic drift.

## 5. Epigenetic Variation as an Evolutionary Mechanism in Natural Populations

**Figure 3:**
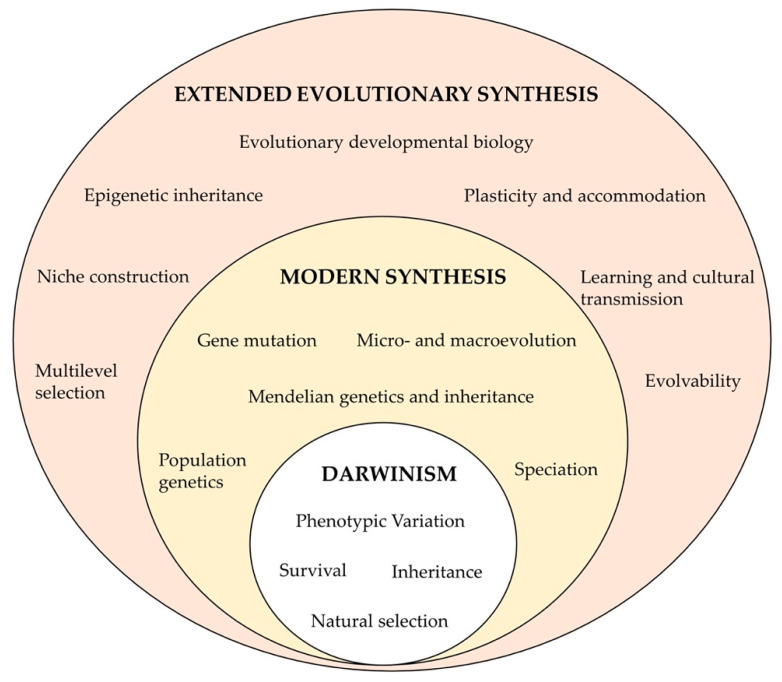
Schematic representation of key concepts included in the Extended Synthesis, illustrating the continuous expansion of evolutionary theories. Adapted with permission from [120], 2022, Massimo Pigliucci.

Natural selection acts on an organism’s phenotypes to enhance their fitness. One of the basic principles of evolution is that phenotypic variation in a population derives from the accumulation of mutations in the DNA sequence which gradually accumulate over generations. However, the slow spread of genetic mutations does not explain all of the observed micro- and macroevolutions, and they cannot keep pace with the rapidly changing environment [66]. Unlike that of genetics, epigenetic inheritance can rapidly affect the population. As described above, epimutations can arise in response to an environmental modification on a much faster time scale, within a single generation than a single de novo genetic mutation in a single individual can. This neo-Lamarckian concept of acquired characteristic inheritance (also known as “soft inheritance”; [119]) and that of the neo-Darwinian evolution should not be seen as incompatible, but instead, they can form a unified theory for evolution which is named the Extended Evolutionary Synthesis (EES) [120]. This theory involves environmentally induced epimutations and an epigenetic transgenerational inheritance that alters the phenotypic variation, on which natural selection can act [121], among other concepts that are illustrated in Figure 3. Several studies on epigenetics in natural animal populations have showed environmentally induced epimutations, but none of them have focused on their evolutionary consequences, which could be the target of future studies. Despite this lack of evidence, the extended evolutionary synthesis theory provides new insights into the microevolution mechanisms for rapid evolutionary events such as an organism’s local adaption to a new environment during introduction and invasive events in a fast-changing habitat, wherein stressors occur intermittently [121], but also, it provides new insights for macroevolution, including radiation and speciation [60]. 

### 5.1. Epigenetics and Microevolution of Natural Animal Populations

Microevolution corresponds to the processes that lead to intraspecific evolutionary changes that occur over time within and among populations. Among these processes, local adaptation is considered to be one of the major mechanisms that is used to explain how organisms adapt to environmental variations or to a new habitat during introduction and invasive events [122]. It is the process by which organisms of the same species evolve and adapt towards different phenotypic optima depending on the local environment in which they live [87]. Studies on plants populations have demonstrated local adaptations that are related to epigenetic variation, close associations between epigenetic variants and environmental gradients in a variety of natural plant systems, and the role of local epigenetic adaptation during biological invasions (e.g., [63,123,124]). These studies have highlighted the importance of epimutations in local epigenetic adaptations. 

Focusing on how epimutations regulate phenotypic traits during local adaptation might shed light on how animal species evolve and which evolutionary strategies they apply. On one hand, environmentally induced epimutations can be associated to a sense-and-response system (i.e., phenotypic plasticity). As described previously in this review, recent studies have showed habitat-specific DNA methylation patterns in a large panel of species. In the previously detailed study of Wogan et al. on the DNA methylation variation in eight populations of the Puerto Rican crested anole *A. cristatellus*, they detected 95 single methylation variants (SMVs), thereby showing a significant relationship to climatic variables: 14 of these were significantly linked to the maximum temperature of the warmest month, and 25 were linked with temperature annual range. Moreover, all of the 95 SMVs were significantly correlated to precipitation seasonality [54]. This study indicates that DNA methylation variation can occur across the environmental gradients of the factors affecting methylation. Just like the plants’ systems, environmentally induced epimutations could play an important role in the phenotypic plasticity, thereby leading to the local adaptation of animals. This strategy seems to be the best if the environmental variations are predictable. Baldanzi et al. investigated the levels of genetic and DNA methylation variation within and among populations of the sandhopper *Talorchestia capensis* from five localities along the South African coasts. Four populations showed significant negative relationships between their epigenetic and genetic diversity (corresponding to a genome-dependent epimutation). The Gansbaai population, the exception, showed no correlation between the two patterns. Interestingly, the Gansbaai population is the only population that is found in a transition area with a high level of environmental changes. Environmentally induced epimutations in the individuals from Gansbaai could be a mechanism of their adaptation to these transitional environmental conditions [50].

On the other hand, random epimutations highlight the propensity to randomly diversify the phenotypes and these are supposed to be more advantageous when organisms encounter unpredictable environmental changes; a strategy that is referred to bet-hedging (i.e., organisms suffering decreased fitness under their normal conditions, but increased fitness under unexpected stressful conditions) [124]. Bet-hedging allows individuals of a population to present a panel of phenotypes including some with high fitness, ensuring the survival of a proportion of the population, whatever the current environmental conditions are. Most incidences of bet-hedging that have been so far highlighted are for prokaryotes, chordates, angiosperms, and arthropods (16 phyla, reviewed in [125]). To our knowledge, the only study in which the methylome of multicellular animals has been studied from a bet-hedging perspective is a study on a wild populations of clonal fish *Chrosomus eos*-*neogaeus* [80]. The authors of this study analyzed the DNA methylation polymorphism in *C. eos-neogaeus* between two types of environment: predictable (lakes) and unpredictable (intermittent streams) areas. They showed that the contribution of environmentally induced and stochastic epigenetic changes strongly differs between the predictable and unpredictable environments. Indeed, clonal fish that are found in predictable environments display environmentally induced epigenetic changes, whereas those living in unpredictable environments are characterized by a high contribution of random epimutations. Thus, pure epigenetic variation (environmentally induced or random) can be adaptive when the environment changes rapidly, thus being predictable or not. 

Epigenetic mechanisms can be associated with another central phenomenon in evolutionary biology and population dynamics: the expansion of newly introduced populations, which is considered as a genetic paradox. These populations succeed when they spread out in a new environment, despite the fact that they are small, presumably not adapted to their new habitat, and encounter a significant decrease in genetic diversity which is associated with passing through a bottleneck [115]. Several natural animal population studies on invasive species have showed that when the genetic diversity is low, epigenetic processes can maintain a high phenotypic variability via a compensatory mechanism between the epigenetic and genetic variation, which could explain their expansion ranges. These studies include observations of the asexual freshwater snail *Potamopyrgus antipodarum* [57,71], the pygmy mussel *Xenostrobus secures*, the tubeworm *Ficopomatus enigmaticus* [49], the mussels *Mytilus galloprovincialis* and *Xenostrobus securis* [126], and the house sparrow *Passer domesticus*. Regarding this latter species, studies have screened for the DNA methylation of the introduced house sparrows in Tampa (Florida) and the Nairobi (Kenya) populations [127], in several cities in Kenya [79], and in the Middle East [128]. Those populations encountered a recent founder effect, thereby reducing their genetic diversity. It turned out that each study obtained the same results: an excess of DNA methylation variation which was relative to genetic variation. Liebl et al. also identified a negative relationship between epigenetic and genetic diversity, which corresponded to a compensatory mechanism for reduced levels of genetic diversity [79]. However, a more recent study on the levels of epigenetic and genetic diversity across 15 sites in the introduced Australian house sparrow population failed to detect any correlation between the two profiles [74]. It suggested that epigenetic diversity is likely to compensate for low genetic diversity that occurs immediately after a bottleneck. A compensatory relationship may have been stronger in the earlier stages of the introduction, but this is now obscured by the genetic diversity recovery. Another insight involves the reversibility of the epigenetic markers. Epigenetic markers are highly dynamic, suggesting that the extent to which DNA methylation signatures are established and removed is variable over time. This study highlights the importance of incorporating history into population-wide epigenetic analysis.

### 5.2. Epigenetics and Macroevolution of Natural Animal Population

Macroevolution corresponds to the processes that lead to interspecific (or higher-rank taxa) evolutionary changes that occur over geologic time. For example, it includes adaptive radiation which is defined by a process in which organisms diversify from an ancestral species into multiple new forms, and this results in speciation. This process particularly occurs when new resources, new environmental niches, or new disturbance arise. In these situations, epigenetic variation is likely to play a role in the initial stages of ecological speciation by facilitating an adaptation to novel ecological environments via phenotypic plasticity. On one hand, a significant environmental shift from a stable habitat to a novel, stable habitat should favour genetic assimilation [129]. During this process, environmental changes induce the epimutations that are responsible for a new advantageous phenotype. This environmentally induced phenotype and its underlying epimutations are maintained across generations and these are subject to natural selection as an adaptive alternative. Over time, these environmentally induced epimutations are incrementally replaced with multiple advantageous genetic mutations through the process of natural selection. The epigenetic contribution to the phenotype decreases as the genetic contribution increases. Ultimately, the environmentally-induced phenotype becomes genetically encoded in the population due to the process of mutation selection, and the environmental signal, as well as the epigenetic marks that are no longer required to produce it [7]. It corresponds to a ‘mutational assimilation’ in which the mutations are facilitated by epigenetics [14]. This process requires that environmentally induced epimutations are inherited through generations and that the environment is stable for a period that is at least as long as the organism’s generation time. This mechanism supports the theory that epigenetic variation precedes genetic variation, as it has the potential to accelerate genetic evolution [11]. On the other hand, a new habitat with fluctuating conditions selects for a high level of plasticity; a process that is named the Baldwin effect [130]. In this case, individuals can express alternative phenotypes due to an alternative methylation pattern established being on some sensitive alleles. These genes that are required for flexibility are selected, and their frequency will increase in the population. In this case, there is no inheritance of the DNA methylation marks. In summary, it is the flexibility of the phenotype that is selected, rather than the result of the flexibility itself. These two concepts of genetic assimilation and the Baldwin effect suggest a role for DNA methylation in the initiation of species divergence and radiation. 

Moreover, field studies have showed that DNA methylation is also involved in the maintenance of species divergence. For example, Skinner et al. compared the epigenetic differences of five closely related species of Darwin’s finches (*Geospiza fortis*, *G. fuliginosa*, *G. scandens*, *Camarhynchus parvulus*, and *Platyspiza crassirostris*) and tested the hypothesis that DNA methylation variation accumulates with phylogenetic distance. They obtained a significant correlation between the number of epigenetic variations and phylogenetic distance between the finches, but no significant between the genetic variants and the phylogenetic distance [65]. This study showed that epimutations appear continuously and accumulate over long periods of time (2–3 Myr). Another study on DNA methylation in fossilized steppe bison *Bison priscus* and bison fresh tissue has showed that there are stable patterns of methylation between ancient and contemporary DNA samples [131]. These findings suggest a role for DNA methylation, not only in the initiation of radiation, but also in the maintenance of species divergence over evolutionary timescales, as epigenetic variations can persist over thousands of generations.

## 6. Future Research in Animal Population Epigenetics

The greatest challenge confronting populations epigenetics is to determine the role of natural epigenetic variation in adaptive evolution. Experimental field studies that are investigating this question in animals are in their first steps. Before considering epigenetics as an evolutionary mechanism, some characteristics have to be investigated or confirmed. First, despite the fact that epigenetic inheritance has been shown in laboratory studies, very few studies have focused on it in wild animal populations [58,118]. Epigenetically induced phenotypes can be transmitted to an organism’s offspring if the epigenetic marks can resist resetting between generations, i.e., epigenetic reprogramming. This mechanism corresponds to an extensive erasing of epigenetic marks, and it occurs both in the germline and in the zygote immediately after fertilization in animals. The reprogramming process has been described in a few species such as mice [132], zebrafish [133], killifish [134], and medaka [135], but we still need to unravel the mechanisms of epigenetic reprogramming in more species in the wild, given its species-specific characteristics. Despite this barrier to transgenerational epigenetic transmission, emerging evidence has shown that pure (random and environmentally induced) epimutation inheritance may exist in animals. Considering the laboratory results, field studies such as those of Wang et al. [58] and Hu et al. [118] would offer a deeper understanding of epigenetic inheritance across individuals under natural conditions, particularly when exploring evolutionary scenarios in wild populations that are facing environmental variation. 

A second feature to investigate is to what extent epigenetic variation is under genetic control. Unfortunately, the correlation between epigenetic and genetic variation in wild animal population studies have not been systematically evaluated. Yet, estimating this correlation is crucial to highlight the evolutionary relevance of epigenetic variation. Regarding studies that have calculated it, there were as many non-significant correlation coefficients as there were significant ones. These results contrast with similar studies in plants that mainly show a strong correlation between the patterns of epigenetic variation and the underlying genetic variants [45]. Otherwise, as genetic variation can blur the role of epigenetic variation, experimental systems in which the confounding effects of genetic variation have been controlled or reduced may be useful for isolating the contributions of epigenetic mechanisms in evolutionary processes. We suggest that future studies could focus on a species with a known limited genetic variation. Researchers have used populations with a lack of genetic variation resulting from clonal reproduction (e.g., clonal fish, [69,70]) or bottlenecks following invasion (e.g., house sparrows, [79,127]). The mixed-mating reproduction system of the mangrove rivulus *Kryptolebias marmoratus* can be used to go even further into the analysis of epigenetic–genetic variations interaction. This system alternates between the cross-fertilization of a male and a hermaphrodite on the one hand, and self-fertilization (or selfing) hermaphrodites on the other hand, which is unique feature among vertebrates [136]. Consistent selfing naturally produces isogenic lineages [137]. Under laboratory conditions, a higher degree of methylation differentiation between genotypes than that between environments has been reported in two highly inbred strains [72]. They also pointed out that methylation differences between environments that are common to both strains mostly correspond to facilitated epialleles, suggesting the existence of a dynamic interaction between the genotype and the environment. For future studies on this species, we suggest the comparison of natural populations that exhibit a selfing rate gradient, and to investigate how epigenetic diversity varies among those populations with a different level of genetic diversity. 

Thirdly, we suggest the use of concepts that have been developed in population genetics studies in their application to population epigenetics, while considering the non-mendelian inheritance and the environmental sensibility of epigenetics. The basic biostatistics of population genetics can be transferred to populations epigenetics to inspire a new index of epigenetic diversity and structure. Johnson and Kelly calculated the *P_ST_*, the methylation analogue of Wright’s *F_ST_*, by subtracting the total variance in the methylation in all populations of Eastern oyster *Crassostrea virginica* from the variance within a single population and divided this by the variance that was in all of the populations (*P_ST_* = (Variance_Total_ − Variance_Sub_)/Variance_Total_) [53]. To take the analysis one step further, characterizing the total epigenetic variation is not sufficient to assess the capacity of an organism to respond to environmental changes. Distinguishing the different types of epimutations (i.e., randomly, genetically, or environmentally induced) might shed light on how organisms evolve in terms of plasticity or diversified bet-hedging adaptations. Field studies could analyse this partition of epigenetic variation as a population characteristic such as those found in population genetics, thereby expanding the molecular tool list to assess the evolutionary potential of populations. 

Fourthly, population epigenetics can be a useful tool in conservation biology. The epigenome can be altered by biotic (e.g., parasitic or social) and abiotic (e.g., thermal or chemical) stressors, thereby creating a permanent epigenetic “foot-print” that is known as epigenetic memory [138]. These environmentally induced DNA methylation patterns can be considered as biomarkers to evaluate the past and present environmental stress events that are experienced by organisms, as there is evidence for epigenetic memory to be transgenerational [139]. To determine the chemical classes to which organisms have been exposed throughout their lifetime using epigenetic memory, more efforts are required in the identification of specific epimutations that are caused by these chemicals. Besides environmental toxicity safety assessments, a DNA methylation variation analysis can be relevant for improving translocations [140] and for studying the connectivity and clustering of wild populations [141]. As such, a DNA methylation study appears to be a promising tool in conservation biology.

Fifthly, the role of DNA methylation in allelic-specific expression (ASE) should be investigated in wild populations. In diploid organisms, genes are generally expressed from both alleles (biallelic expression), but there are exceptions wherein it occurs only from one allele (monoallelic expression), thereby creating an ASE at each involved gene locus. An ASE is the consequences of an epigenetic process that silences one of the parentally inherited alleles of a gene [142]. The most well-known examples of an ASE that is mediated by epigenetic mechanisms are genomic imprinting and X-chromosome inactivation [143]. Interestingly, random monoallelic expressions (RME) can also occur at the individual loci of autosomal genes, independently of the gene families [144]. Studies have showed that RME patterns are inherited during cell division [145,146], meaning that the earlier that this process occurs during development, the more cells and tissues that will express similar ASEs, and vice versa. This stochasticity that is provided by RME generates a wide diversity of gene expression and might confer many advantages such as generating cellular diversity or regulating gene dosage, as is observed in X-chromosome inactivation. As some cells could have advantageous combination of ASE patterns, it can also enhance the adaptability of organisms to environmental changes during development and throughout their life. Thus, the epigenetic regulation of allelic-specific expressions could create an expression imbalance that contributes to the generation of phenotypic variation among individuals. 

Finally, regarding future research on animal populations, epigenetics complexity is worth noting. As detailed in the introduction, the genomic distribution of DNA methylation has been found in many clades of animals, but there are differences in how and where it occurs. Moreover, it is well established that different tissues have specific DNA methylation patterns within the organism, that there is an epigenetic dysregulation with age, and also an interaction between these two criteria as some studies show a tissue-specific effect of age on the epigenome [147,148]. A comparison of the studies characterizing DNA methylation diversity should therefore be interpreted with caution, although it is necessary to draw general lines on epigenetic variation in natural populations. Epigenetics multiplicity is also worth noting; while DNA methylation is the main studied epigenetic mechanism, RNA interference and histone modification are further mechanisms that must be included in the discussion about gene expression regulation generating phenotypic diversity.

## 7. Conclusions

DNA methylation diversity has been found to be a revealing parameter to characterize natural animal populations. Further studies on its dynamics, emergence, and subsequent implications in population fitness has become increasingly relevant, especially from evolutionary perspectives. The recent progress in ecological epigenetics allows a more complete understanding of how epigenetic diversity is modulated over time, which will be helpful for generating predictive models of the capacity of populations to adapt to environmental variation. Distinguishing random epimutations from environmentally induced ones and heritable epimutations from non-heritable ones may allow us to characterize the responses of organisms to environmental changes, as any variations in DNA methylation within a species might shed light on how they evolve. Although epigenetic studies in natural animal populations are relatively scarce and new, they highlight some important characteristics of DNA methylation that can be used in future research to investigate the link between epigenetic variation and phenotypic plasticity, and local adaptation and evolutionary mechanisms in the wild.

## Figures and Tables

**Table 1:** Overview of studies focusing on genetic and epigenetic diversity and correlation in natural animal populations.

Species	Genetic vs. Epigenetic Correlation	Epialleles Category	Ref.
Clonal fish (*Chrosomus eos-neogaeus*)	No significant correlation	Putatively pure or facilitated	[69]
Clonal fish (*Chrosomus eos-neogaeus*)	No significant correlation	Putatively pure or facilitated	[70]
Clonal fish (*Chrosomus eos-neogaeus*)	No significant correlation	Unknown	[80]
House sparrows (*Passer domesticus*) (Africa)	Significant negative correlation	Unknown	[79]
House sparrows (*Passer domesticus*) (Australia)	No significant correlation	Unknown	[74]
Red grouse (*Lagopus lagopus scotica*)	No significant correlation	Unknown	[81]
Bats (*Rhinolophus pusillus*, *Hipposideros armiger* and *Miniopterus fuliginosus*)	Significant positive correlation	Unknown	[66]
South African (Gansbaii) sandhopper (*Talorchestia capensis*)	No significant correlation	Putatively pure or facilitated	[50]
South African sandhopper (*Talorchestia capensis*)	Significant negative correlation	Putatively obligatory	[50]
Pacific oyster (*Crassostrea gigas*)	Significant positive correlation	Putatively obligatory	[67]
Crested anole (*Anolis cristatellus*)	Significant positive correlation	Putatively obligatory	[54]
Eastern oyster (*Crassostrea virginica*)	No significant correlation	Unknown	[53]
Fish (*Gobio occitaniae*)	Significant positive correlation	Unknown	[75]
Chinook salmon (*Oncorhynchus tshawytscha*)	No significant correlation	Unknown	[82]

## Data Availability

Not applicable.

## Appendix A

**Table A1:** Glossary.

Term	Definition
Epigenetic mark	Chemical modifications to DNA, RNA, or proteins that influence chromatin state and gene expression. It includes DNA methylation, noncoding RNAs, and protein modifications (e.g., acetylation, deacetylation, ubiquitination, and histone methylation).
Epiallele	Locus presenting distinct epigenetic profiles due to differences in methylation or chromatin states.
Epimutation	Heritable change in gene activity that is not associated with a DNA mutation, but rather with the gain or loss of DNA methylation or other heritable modifications of chromatin.
Single methylation polymorphism (SMP)	Spontaneous variation in DNA methylation at base-pair resolutions that are due to errors in the maintenance of methylation states. The rates of SMP formation is at least four orders of magnitude greater than genetic mutations.
Epigenetic reprogramming	Erasure and remodelling of epigenetic marks such as DNA methylation during embryo development. Its purposes include the erasure and reestablishment of parental genomic imprints in germ cells, the erasure of epimutations, and the correct development of the embryo through the generation of totipotent or multipotent cells.
Epigenetic variation	Variation in epialleles which is studied among and/or within populations.
Epigenetic diversity	The amount of epigenetic variation within a population.
Transgenerational epigenetic inheritance (TEI)	Stable inheritance of epigenetic marks across multiple generations.
Epigenetic divergence	The process in which two or more populations of an ancestral species accumulate independent epimutations through time.
Epigenetic drift	Gradual changes in the epigenome that is due to random epimutations. This neutral process is not directional as it creates ﻿both hyper- and hypomethylation.
CpG island	Short CpG-rich region of the genome characterized by at least 500 bp of DNA with a GC content ≥ 55%.
Epigenetic potential	The genomic capacity for environmentally induced phenotypic change (i.e., plasticity) via epigenetic modifications.
Phenotypic plasticity	Any change in an organism’s phenotype in response to an environmental signal.
Neo-Lamarckism or Lamarckian inheritance	A theory of evolution based on the principle of soft inheritance, which refers to the inheritance of variations that are the result of non-genetic effects. It includes inheritance coming from evolutionary developmental biology, epigenetics, niche construction, and learning and cultural transmission. This theory is part of the extended evolutionary synthesis.
Extended evolutionary synthesis	A set of evolutionary theories including the modern synthesis (combination of Darwinian view of evolutionary change and Mendelian genetics) and soft inheritance (or Lamarckian inheritance). This theory is still under debate.

**Table A2:** Commonly used molecular techniques to evaluate DNA methylation diversity in field studies.

Technique	Description, Advantages, and Limitations
Methylation-sensitive amplified polymorphism (MSAP)	Modified from the amplified fragment length polymorphism (AFLP) technique, MSAP uses *Eco*RI (rare cutter) with either one or two methylation-sensitive isoschizomer restriction enzymes, *Hpa*II and *Msp*I (frequent-cutter), which recognize the same restriction site (5′-CCGG-3′) but have different cytosine methylation sensitivities. For each sample, MSAP analysis is performed using both *Eco*RI/*Hpa*II- and *Eco*RI/*Msp*I-digested samples. The resulting DNA fragments are ligated with linkers and PCR amplified. Such amplification produces a reduced population of fragments that are separated in denaturing polyacrylamide gels in order to compare the respective band patterns. This technique is useful for non-model species as it does not require a reference genome. It is one of the most commonly used methods for assessing DNA methylation changes in plants. However, the main disadvantage of MSAP is that it can only detect methylation on 5′-CCGG-3′.
Methylated DNA immunoprecipitation (MeDIP)-Seq	MeDIP is an enrichment-based purification technique that involves antibodies directed against mC or mCG to precipitate methylated DNA fragments. Differential DNA methylation regions are identified by comparing the coverage between groups of interest. Combining MeDIP with next-generation sequencing, it provides methylomes at typically 100-bp to 300-bp resolution. With the appropriate antibody, MeDIP is also able to detect hmC. MeDIP limitations include antibody quality and cross-reactivity, and relatively low-resolution level in comparison with bisulfite sequencing methods.
Reduced representation bisulfite sequencing (RRBS)	RRBS relies on digestion of genomic DNA with the enzyme MspI, which produces DNA fragments that begin and/or end with an informative CpG site (CpG-enriched genomic regions). Then, genomic DNA is treated with sodium bisulfite, which leaves methylated cytosines intact but converts unmethylated cytosines to uracil (and ultimately thymine after PCR). Amplification fragments are sequenced, allowing for the identification of methylated cytosines. This is an efficient and high-throughput technique due to its high definition since it produces genome-wide methylation profiles with single-nucleotide resolution. Compared to WGBS, it allows one to investigate larger numbers of individuals as it is more cost-effective, but it only provides limited genome coverage (5–10%) and is CpG island and promoter region-centric.
Whole-genome bisulfite sequencing (WGBS	WGBS combines the use of sodium bisulfite treatment and high-throughput DNA sequencing to produce genome-wide methylation profiles with single-nucleotide resolution. Unlike RRBS, it estimates all cytosines methylation levels (including CpG and non-CpG) across the genome, rather than CpG enriched genomic regions. This method is capable of testing approximately 90% of all cytosines in genomes studied to date but is cost prohibitive to sequence large numbers of individual samples.
